# Effects of angiotensin receptor blockers on neointimal characteristics in angina patients requiring stent implantation: optical coherence tomography analysis

**DOI:** 10.1186/s12872-017-0709-9

**Published:** 2017-11-15

**Authors:** Jae Young Cho, Soon Jun Hong, Do-Sun Lim

**Affiliations:** Department of Cardiology, Korea University Anam Hospital, Inchon-ro 73, Seongbuk-gu, Seoul, South Korea

**Keywords:** Angiotensin receptor blockers, Neointima, Drug-eluting stents, Optical coherence tomography

## Abstract

**Background:**

Angiotensin receptor blockers (ARBs) are known for its anti-inflammatory and anti-proliferative effects. The aim of the study was to evaluate long-term effects of ARBs on morphologic characteristics of stent restenosis in patients with coronary artery disease requiring stent implantation by optical coherence tomography (OCT).

**Methods:**

Patients with coronary artery disease having history of drug-eluting stent implantation (*n* = 407) were analyzed on the basis of ARB therapy as the ARB group (*n* = 162) and the non-ARB group (*n* = 245). Neointimal characterizations were performed at lesions with diameter stenosis >30% with OCT in each group. Major adverse cardiovascular events (MACEs), lumen area, stent area, neointimal area, neointimal thickness, nonapposed struts, uncovered struts, and intraluminal mass between two groups were also observed.

**Results:**

More patients in the ARB group revealed homogeneous and layered neointimal pattern (44.9% vs. 35.6%, *P* < 0.001, and 16.8% vs. 10.6%, *P* < 0.001, respectively), and whereas patients in the non-ARB group revealed heterogeneous neointimal pattern (1.1% vs. 7.6%, *P* < 0.001). Mean neointimal area (1.09 ± 1.00 mm2 vs. 1.38 ± 1.24 mm2) and mean neointimal thickness (140.6 ± 112.0 μm vs. 189.6 ± 423.1 μm) with OCT were smaller in the ARB group when compared to the non-ARB group. Percentage of covered stents was significantly higher in the ARB group when compared to the Non-ARB group (97.3% vs. 92.6%, *P* = 0.015). Other factors such as follow-up % diameter stenosis, late lumen loss, binary restenosis, MACEs, various neointimal characteristics analyzed by image analyzing software did not show significant differences.

**Conclusion:**

The use of ARBs after drug-eluting stent implantation demonstrated difference in neointimal characteristics, less amount of neointimal area and fewer number of uncovered stent struts during the follow-up OCT, indicating the anti-proliferative and anti-inflammatory effects of ARBs.

## Background

Angiotensin receptor blockers (ARBs) have been widely used in hypertensive patients with coronary artery disease (CAD) requiring stent implantation. In addition to its antihypertensive effect, ARBs are also known for their anti-inflammatory and anti-proliferative effects. The renin–angiotensin system has been implicated in the pathogenesis of restenosis and acute coronary syndrome [1–6] and, thus, may be a potential target for the prevention of in-stent restenosis and atherothrombotic events in patients who have CAD. It is well known that direct vascular effects of angiotensin II include vasoconstriction, inflammation, endothelial dysfunction, and stimulation of growth processes and remodeling, which are mediated by type 1 receptors [3, 7]. The single-center VALsartan for Prevention of REstenosis after Stenting of Type B2/C lesions (VAL-PREST) trial documented the remarkable therapeutic effects of the ARB valsartan on restenosis after stenting in complex coronary lesions [8]. In that trial, the ARB was more effective than placebo for preventing in-stent intimal proliferation and its superiority over an angiotensin-converting enzyme (ACE) inhibitor can be predicted from the result that 68% of patients in the placebo group were taking an ACE inhibitor.

Optical coherence tomography (OCT) is an emerging intracoronary diagnostic modality that provides high-resolution images of coronary artery in vivo [9]. In addition to tissue characterization in native coronary plaques, OCT has been applied to characterize neointima after stent implantation [10]. Indeed, several OCT studies revealed the development of lipid-laden neointima inside the stents, and OCT has become the modality of choice to study atherosclerotic change of neointima [11–13]. However, correlation between anti-proliferative effect of ARB and change of neointimal characterization has not been studied yet. We investigated and compared the long-term effects of ARBs on neointimal characteristics in patients with CAD requiring stent implantation by OCT image analysis.

## Methods

### Study design and patients

This study was based on a retrospective observational data on patients who underwent OCT in Korea university Anam hospital between January 2011 and December 2012. Patients with CAD having history of drug-eluting stent (DES) implantation were analyzed on the basis of ARB therapy. Type of ARB or DES was not limited and administering other medications such as aspirin, β-blocker, calcium channel blocker for angina was not prohibited. Patients were divided in terms of taking ARBs as the ARB group and the non-ARB group. Inclusion criteria were: age between 40 to 75 years, CAD requiring drug-eluting stent implantation, diagnosed with hypertension or under antihypertensive medications, and OCT measurements during the follow-up. Patients with left main CAD, previous history of coronary artery bypass graft, acute myocardial infarction (MI), chronic total occlusion, ejection fraction <50%, unsuccessful reperfusion after coronary stent implantation, and liver or renal dysfunction were excluded from this study. Age and risk factor matching (diabetes, hyperlipidemia, smoking, family history of CAD) was performed. Major adverse cardiovascular events (MACEs) such as all-cause death, non-fatal MI, stroke, and target lesion revascularization (TLR) were compared between the two groups during the follow-up.

### Quantitative coronary angiography

Offline quantitative coronary angiography (QCA) was conducted using the view that revealed the highest degree of stenosis. Severity of coronary stenosis was measured using the Cardiovascular Measurement System (MEDIS Medical Imaging System; Leiden, The Netherlands). For every patient, angiograms were analyzed at the time of OCT examination. Lesion length, reference diameter, minimal luminal diameter, and percent diameter stenosis were calculated by a single operator who was blinded to clinical characteristics. Analysis of angiographic frames was performed in the end-diastolic stage. Angiographic restenotic lesion type was classified as follows: focal restenosis, <10 mm in length (A) (articulation or gap [IA], margin [IB], focal body [IC], multifocal [ID]), or diffuse intrastent restenosis (B), >10 mm in length (intrastent [II], proliferative [III]) [14].

### OCT examination and analysis

OCT examination and analysis was performed during the follow-up (LightLab Imaging Inc., Ilumien Offline review workstation, Ver D.O 2, MA, USA). Under guidance of a 0.014 in. angioplasty wire, OCT imaging catheter (C7 DragonflyTM, LightLab Imaging Inc., MA, USA) was advanced into the distal end of the DES implantation site. The entire length of the stent was imaged with an automatic pullback device moving at 15 mm/s and the OCT image clearly visualized the stent cross-section.

**Fig. 1:**
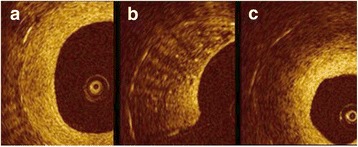
Neointimal characterizations. **a** Homogeneous **b** Heterogeneous **c** Layered

**Fig. 2:**
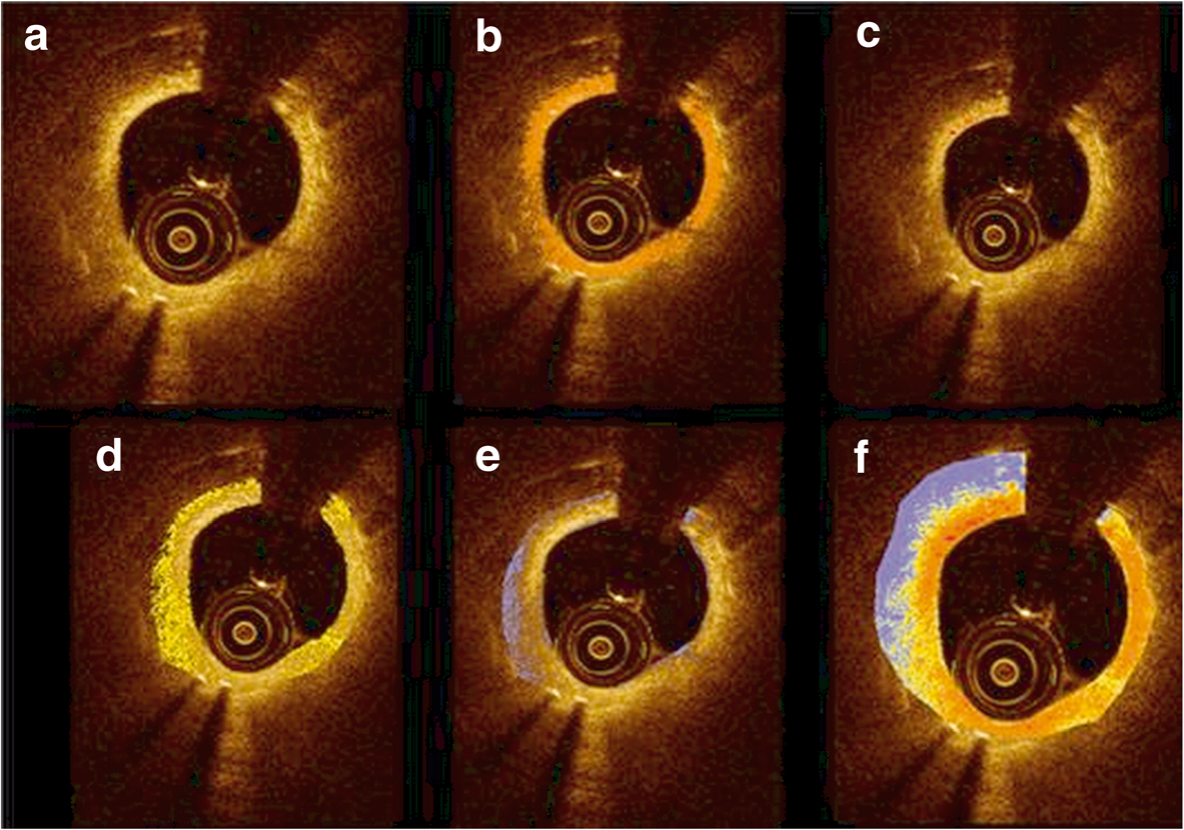
Neointimal characterizations categorizing by contrast-color matching. **a** Original OCT finding **b** Macrophage or cholesterol crystal **c** Fibrous plaque **d** Proteoglycan plaque **e** Lipid or calcified plaque or neovascularization **f** Whole image of analysis

**Fig. 3:**
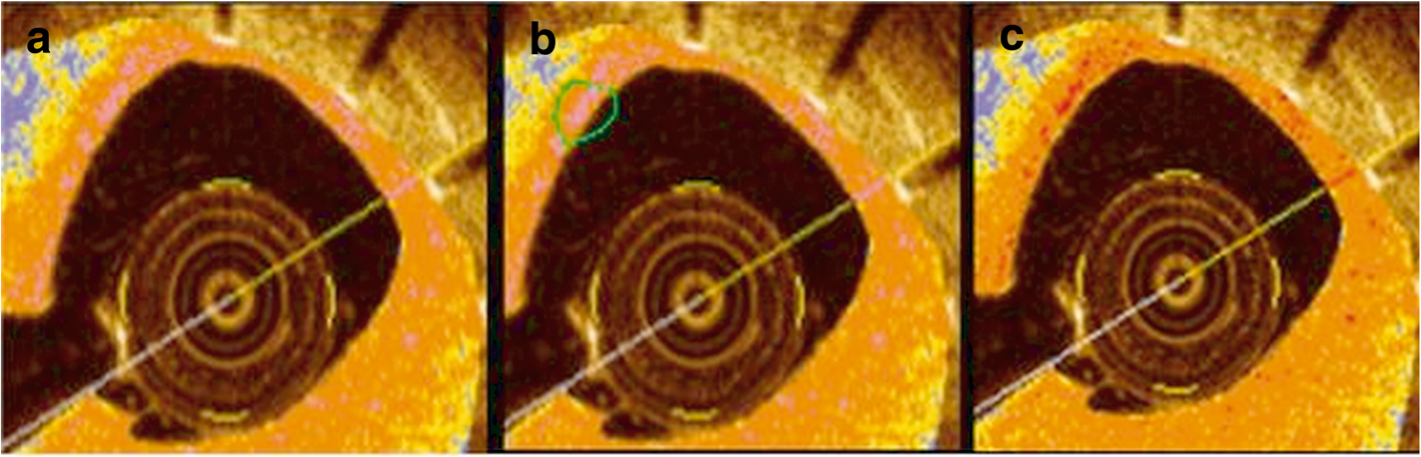
Neointimal characterizations categorizing by contrast-color matching. **a** original OCT finding filled with contrast-color category **b** Count whole contrast-color pixels (color pink) **c** Set boundary of corresponding lesion (green)

Neointimal characterizations were performed with OCT images at lesions of diameter stenosis >30% and were analyzed into two groups with same lesions by experienced observers using established criteria [10, 15]. The first group was categorized simply with patterns defining neointimal tissue structure of homogeneous, heterogeneous or layered (Fig. 1). Another group was classified into seven categories using densitometric analysis with image analyzing software (Image Pro Plus 7.0, Media cybernetics Inc., Bethesda, MD, USA): (1) macrophage, (2) cholesterol plaque, (3) fibrous plaque, (4) proteoglycan rich plaque, (5) calcified plaque, (6) lipid plaque, and (7) neovascularization. With densitometry, ranging from contrast of minimum 0(darkest) to maximum 255(brightest), lipid or calcified plaque or neovascularization was set to 0–69 (purple), proteoglycan plaque to 70–108 (yellow), fibrous plaque to 109–194 (orange) and macrophage or cholesterol crystal to 195–255 (red) (Fig. 2). Overlapping categories (macrophage or cholesterol crystal and lipid plaque or calcified plaque or neovascularization) could not be differentiated by densitometry; therefore, setting boundaries manually between overlapping categories and calculating ratio with pixels was done manually (Fig. 3). Lumen area, stent area, neointimal area, neointimal thickness, nonapposed struts, uncovered struts, and intraluminal mass were also observed.

### Study endpoints

Primary end point was to compare neointimal characteristics and neointimal area with OCT during the follow-up. The secondary end point was to compare major adverse cardiovascular events (MACEs defined as non-fatal MI, death, stroke, and TLR) and late lumen loss, diameter stenosis, in-stent restenosis (defined according to the Academic Research Consortium criterion) [16] during the follow-up.

### Statistical analysis

Categorical variables are expressed as numbers and percentages. Continuous variables are expressed as mean ± SD. Comparisons between groups were performed with 2-tailed Student t test for continuous variables and with χ2 or Fisher exact test for categorical variables. The reproducibility of qualitative variables was assessed with κ test. A *P* < 0.05 was considered statistically significant. All statistical analyses were performed using SPSS (SPSS 12.0; Chicago, IL) software.

## Results

### Participant characteristics and baseline assessments

In total, we included 407 patients who were diagnosed as CAD having history of DES implantation and underwent OCT. Of these patients, 162 (39.8%) were in ARB group and 245 (60.2%) were in non-ARB group. Patient baseline characteristics were well matched in the study groups except sex. In the ARB group, fewer patients were male compared with the non-ARB group (66.0% vs. 79.6%, *P* = 0.002). Regarding as a retrospective data, various types of ARB were used in this study but difference of other medication use for angina was not statistically significant except CCB (24.1% vs. 68.9%, *P* < 0.001) (Table 1).

**Table 1:** Baseline patient characteristics

Variable	ARB Group (*n* = 162)	Non-ARB Group (*n* = 245)	*P* value
Age (years)	60.8 ± 9.5	60.5 ± 10.1	0.751
Men	107 (66.0%)	195 (79.6%)	0.002
Body mass index (kg/m^2^)	24.7 ± 4.3	24.9 ± 4.2	0.372
Risk factors			
Diabetes	55 (34.0%)	68 (28.1%)	0.21
Hyperlipidemia	64 (39.5%)	101 (41.2%)	0.119
Current smoker	38 (23.9%)	67 (27.7%)	0.208
Family History of CAD	4 (2.5%)	5 (2.1%)	0.766
History of CVA	3 (1.9%)	4 (1.6%)	1
LVEF (%)	55 ± 5	54 ± 4	0.673
Mean follow-up duration (months)	19.0 ± 11.4	20.3 ± 14.1	0.523
ARBs			
Candesartan	34 (21.0%)		
Valsartan	29 (17.9%)		
Irbesartan	34 (21.0%)		
Telmisartan	10 (6.2%)		
Losartan	31 (19.1%)		
Olmesartan	15 (9.3%)		
Eprosartan	10 (6.2%)		
Medications			
Aspirin	160 (98.8%)	235 (95.9%)	0.859
Clopidogrel	106 (65.4%)	169 (69.0%)	0.582
Statins	153 (94.4%)	237 (96.7%)	0.775
Beta blocker	34 (21.0%)	51 (20.8%)	0.218
Calcium channel blockers	39 (24.1%)	169 (68.9%)	<0.001
Diuretics	17 (10.5%)	27 (11.0%)	0.947

*CAD* coronary artery disease, *CVA* cerebrovascular accident, *LVEF* left ventricular ejection fraction, *ARBs* angiotensin receptor blockers

Values are expressed as mean ± SD for quantitative variables or as n (%) for qualitative variables

### Target lesion characteristics and QCA analysis

Target arteries or types of target lesion did not differ within both groups (Table 2). Atherosclerotic lesions were located in left anterior descending artery (LAD) mainly (72.8% vs. 68.7%, *P* = 0.129), and angiographic restenotic lesion type C took the largest portion in both groups (44.1% vs. 43.3%, *P* = 0.939). Although several types of DES used in the study, there was no significant difference. Also, QCA findings on baseline, postprocedure and OCT follow-up revealed no significant differences in any parameters between the 2 groups (Table 3).

**Table 2:** Target lesion characteristics

Variable	ARB Group (*n* = 162)	Non-ARB Group (*n* = 245)	*P* value
Number of lesions stented	195	307	
Target coronary artery	142 (72.8%)	211 (68.7%)	
LAD	31 (15.9%)	49 (16.0%)	0.129
LCX	22 (11.3%)	47 (15.3%)	0.91
RCA			0.104
Type of lesion (%)	5 (2.6%)	11 (3.6%)	
A	35 (17.9%)	56 (18.2%)	0.636
B1	69 (35.4%)	107 (34.9%)	0.877
B2	86 (44.1%)	133 (43.3%)	0.945
C	121 (62.1%)	175 (57.0%)	0.939
Stent type			
EES	105 (64.8%)	147 (60.0%)	0.288
BES	29 (17.9%)	47 (19.2%)	0.768
ZES	16 (9.9%)	32 (13.1%)	0.342
SES	11 (6.8%)	20 (8.2%)	0.622

*LAD* left anterior descending artery, *LCX* left circumflex artery, *EES* everolimus-eluting stent, *BES* Biolimus A9-eluting stent, *ZES* zotarolimus-eluting stent, *SES* sirolimus-eluting stent

Values are expressed as mean ± SD for quantitative variables or as n (%) for qualitative variables

**Table 3:** QCA Measurements at baseline

Variable	ARB Group (*n* = 162)	Non-ARB Group (*n* = 245)	*P* value
Baseline			
RD (mm)	2.54 ± 0.21	2.58 ± 0.16	0.454
MLD (mm)	0.63 ± 0.25	0.58 ± 0.31	0.323
% stenosis	75 ± 6	78 ± 8	0.401
Mean lesion length (mm)	19.5 ± 8.5	22.2 ± 10.4	0.287
Postprocedure			
RD (mm)	2.65 ± 0.28	2.68 ± 0.32	0.128
MLD (mm)	2.53 ± 0.27	2.52 ± 0.29	0.323
% stenosis	5 ± 4	6 ± 4	0.709
Acute gain (mm)	1.9 ± 0.3	1.9 ± 0.4	0.892
Mean stent length (mm)	23.1 ± 7.9	25.0 ± 7.5	0.564
Mean stent diameter (mm)	2.64 ± 0.37	2.65 ± 0.36	0.768

*QCA* quantitative coronary angiography, *RD* reference diameter, *MLD* minimal lumen diameter

Values are expressed as mean ± SD for quantitative variables or as n (%) for qualitative variables

### Endpoints

Descripted above, neointimal characteristics obtained by OCT were analyzed in two different methods, three patterns of homogenous, heterogeneous or layered and seven categories of using image analyzing software. As we analyzed with former method, all three patterns were differed with statistical significance between two groups (Table 4). Homogeneous and layered pattern existed more in the ARB group (44.9% vs. 35.6%, *P* < 0.001 and 16.8% vs. 10.6%, *P* < 0.001, respectively), whereas heterogeneous pattern was more seen in the non-ARB group (1.1% vs. 7.6%, *P* < 0.001). By using latter method, both groups were statistically similar (Table 4), but macrophage seemed to be lower in the ARB group (0.98% vs. 1.23%, *P* = 0.225). Secondary endpoints consist with comparing major adverse cardiovascular events and late lumen loss, diameter stenosis, in-stent restenosis during the follow-up did not show any statistical significance, although patients in the ARB group were more likely to occur TLR than the non-ARB group (15.4% vs. 9.4%, *P* = 0.077). In quantitive OCT findings, stent struts were covered more likely in the ARB group than the non-ARB group (97.3% vs. 92.6%, *P* = 0.015) (Table 5).

**Table 4:** Primary endpoint. Neointimal characterization by OCT

	ARB Group (*n* = 162)	Non-ARB Group (*n* = 245)	*P* value
Analyzing patterns			
Number of lesions analyzed	89	132	
Homogenous pattern	40 (44.9%)	47 (35.6%)	<0.001
Layered pattern	15 (16.8%)	14 (10.6%)	<0.001
Heterogeneous pattern	1 (1.1%)	10 (7.6%)	<0.001
Analyzing with Image Pro			
Number of lesions analyzed	57	81	
Macrophage (%)	0.98	1.23	0.225
Cholesterol plaque (%)	0.01	0.00	0.355
Fibrous plaque (%)	55.63	58.36	0.479
Proteoglycan rich plaque (%)	26.4	25.7	0.705
Calcified plaque (%)	0.21	0.11	0.568
Lipid plaque (%)	16.73	13.28	0.198
Neovascularization (%)	0.04	0.19	0.217

*OCT* optical coherence tomography

Values are expressed as mean ± SD for quantitative variables or as n (%) for qualitative variables

**Table 5:** Secondary endpoint. Cardiovascular events, QCA measurements and quantitative OCT findings during the follow-up period

Cardiovascular events	ARB Group (*n* = 162)	Non-ARB Group (*n* = 245)	*P* value
Cardiovascular Events			
Non-fatal MI	3 (1.9%)	2 (0.8%)	0.354
Cardiac death	1 (0.6%)	0	0.319
Stroke	0	0	1.000
TLR	25 (15.4%)	23 (9.4%)	0.077
QCA measurements			
Angiographic follow-up duration (months)	19.0 ± 11.4	20.3 ± 14.1	0.523
RD (mm)	2.66 ± 0.30	2.68 ± 0.34	0.543
MLD (mm)	2.24 ± 0.29	2.21 ± 0.27	0.648
% stenosis	16 ± 8	19 ± 12	0.321
Late lumen loss (mm)	0.29 ± 0.28	0.32 ± 0.36	0.196
Binary restenosis	14 (8.6%)	22 (9.0%)	0.465
Quantitative OCT findings			
Mean lumen area, mm^2^	6.40 ± 2.49	6.74 ± 2.18	0.318
Mean neointimal area, mm^2^	1.09 ± 1.00	1.38 ± 1.24	0.096
Mean neointimal thickness, μm	140.6 ± 112.0	189.6 ± 423.1	0.217
Malapposed strut No, %	0.70%	1.00%	0.322
Exposed strut No, %	0.80%	1.10%	0.372
Covered strut No, %	97.30%	92.60%	0.015

*MI* myocardial infarction, *TLR* target lesion restenosis, *QCA* quantitative coronary angiography, *RD* reference diameter, *MLD* minimal lumen diameter, *OCT* optical coherence tomography, *No* numbers

Values are expressed as mean ± SD for quantitative variables or as n (%) for qualitative variables

## Discussion

Stent restenosis is an infrequent but poorly understood clinical problem in the drug-eluting stent era and its treatment is challenging. Experimental and clinical studies have identified excessive neointimal hyperplasia as leading cause of stent restenosis [17–19]. Neointima was composed of various characteristic lesions, such as fibrous tissue, proteoglycan-rich tissue, approved by pathologic examination of restenosis in bare-metal stent (BMS) and in DES in tissue samples obtained by atherectomy [20]. In the other hand, ARBs inhibit atherosclerosis as reduced plaque burden in atherosclerotic vessels and reduced incidence of in-stent restenosis [21]. In this concept of anti-inflammatory and anti-proliferative effect of ARBs toward atherosclerosis and neointimal growth, our present study assessed difference of neointimal characterization in use of long term ARBs by OCT. Furthermore, we tried to discover changes of specific neointimal component related to ARBs use.

Neointimal characterization in OCT was analyzed into two groups: a group which is classified conventionally into three patterns of homogeneous, heterogeneous and layered, and another group of specific neointimal component described above in methods. Considering former group, although currently no OCT criteria have been validated with histology for the identification of these tissue types, there are consumptions of each pattern correlating with neointimal characteristics. Homogeneous pattern is regarded identical to fibrous plaque, which was shown predominance in DES comparing with BMS [20]. Heterogeneous pattern is believed to be a mixture of various neointimal components within whole portion of neointimal growth that might result to show different optical properties [10]. Layered pattern could be visualized in OCT based on pathologic examinations that have demonstrated the density and orientation of smooth muscle cells vary within restenotic tissue compared to the inner luminal border and tissue located far from the lumen [22]. In the current study, homogeneous and layered pattern of neointima were more likely to be present in use of ARBs. Considering an experimental data that ARBs increased the thickness of the fibrous cap and collagen content in the plaque resulting in reduce of plaque vulnerability [23] and homogeneous pattern is identical to fibrous plaque, our result suggests neointimal change seen in OCT by ARBs may have correlation histopathology. As layered pattern is known to be divided into the inner luminal border, which the smooth muscle cells are more compact and show a homogeneous concentric orientation, whereas the density of cells decreases and the orientation becomes more heterogeneous in the tissue located far from the lumen, it can be assumed that stabilized neointima by ARBs can be explained as homogeneous portion. In contrast, heterogeneous pattern was more appeared in the non-ARB group. This can mean in an opposite view that anti-inflammatory effect of ARBs play a role for less appearing of heterogeneous pattern in long-term ARBs use.

Another goal was to objectify and quantify specific neointimal component by using image analyzing software, so as to classify lesions easily. However, an attempt to discover changes of specific neointimal component related to ARBs use has failed, although macrophage seemed to be lower in the ARB group (0.98% vs. 1.23%, *P* = 0.225), but in other hand, recent study showed decreased plaque macrophages after use of ARBs in histological evaluation [23]. Main cause of the difference between histological finding and result OCT analysis by densitometry can be thought as the measurement bias, as lesions were defined not by characteristic morphology but brightness.

Interestingly, stent struts were covered more likely in the ARB group than the non-ARB group (97.3% vs. 92.6%, *P* = 0.015). Degree of reendothelialization, which is identical with coverage of stent struts, is regarded as the most powerful predictor of stent thrombosis proven by an autopsy or animal study [24, 25]. ARBs attenuate in-stent neointima formation by inhibiting inflammation and progenitor cells, and in other hand, angiotensin II is known to exert an inhibitory effect on thrombolysis, stimulating thrombus formation, suggesting that ARBs may have an anti-thrombotic effect also [26, 27]. Contradiction of opposing effects of ARBs may have caused the situation that there was no difference of cardiovascular event including thrombotic diseases between the ARB and non-ARB group.

### Study limitations

Present study has several limitations. Unlike prior studies that revealed ARBs reduced incidence of in-stent restenosis, difference of TLR between two groups in our result did not show any statistical significance. It may be because of the retrospective character, the limited sample size, or different types of ARBs were used in this study. Available data suggest that the beneficial effects of ARBs on atherosclerosis cannot be considered a class effect. Losartan and candesartan have not shown any effect on atherosclerosis or on in-stent restenosis, but valsartan, olmesartan and telmisartan appear to have a significant beneficial effect [21]. Also, various types of DES were used, from first generation (SES) to second generation (EES, BES, ZES), limiting this study that second-generation DES lead to a lower percentage of uncovered and malapposed struts, as well as a lower incidence of intra-stent thrombi, compared with first-generation DES [28, 29]. Measurement bias in analyzing densitometry with image analyzing software was described above in discussion.

## Conclusions

In conclusion, the use of ARBs after DES implantation demonstrated different neointimal characteristics when compared with the non-ARB group, and stent struts were covered more likely with the use of ARBs during the follow-up OCT, indicating the anti-inflammatory effects of ARBs. For more definite conclusions, long-term clinical and serial OCT follow-up with a larger population will be needed in the future.

## Abbreviations

ACE: Angiotensin converting enzyme
ARB: Angiotensin receptor blocker
BMS: Bare-metal stent
CAD: Coronary artery disease
DES: Drug-eluting stent
MACE: Major adverse cardiovascular event
OCT: Optical coherence tomography
QCA: Quantitative coronary angiography
TLR: Target lesion revascularization
VAL-PREST: Valsartan for prevention of restenosis after Stenting of Type B2/C lesions trial
